# Mind the (transition) gap: Youth mental health-oriented early intervention services to overcome the child-adolescent vs. adult hiatus

**DOI:** 10.3389/fpsyt.2022.965467

**Published:** 2022-08-17

**Authors:** Michele Poletti, Antonio Preti, Andrea Raballo

**Affiliations:** ^1^Department of Mental Health and Pathological Addiction, Child and Adolescent Neuropsychiatry Service, Azienda USL-IRCCS di Reggio Emilia, Reggio Emilia, Italy; ^2^Department of Neuroscience, University of Turin, Turin, Italy; ^3^Section of Psychiatry, Clinical Psychology and Rehabilitation, Department of Medicine, University of Perugia, Perugia, Italy; ^4^Center for Translational, Phenomenological and Developmental Psychopathology (CTPDP), Perugia University Hospital, Perugia, Italy

**Keywords:** clinical high-risk for psychosis, early intervention services, homotypic transition, heterotypic outcomes, youth mental health

## Introduction

Two decades of worldwide implementation of early intervention services (EIS), inspired by the clinical staging model of psychosis and the related Clinical High-Risk for Psychosis (CHR-P) operational syndromes, have recently sparkled a vivid debate on pro & cons of EIS themselves.

The main critiques concern:

1. The apparent poor predictive specificity of the CHR-P paradigm with respect to longitudinal outcomes, particularly transition to psychosis. Meta-analytical reports indeed suggesting that only about one-third of at-risk subjects undergo a psychometric transition to psychosis, whereas almost two-thirds do not remit from psychological suffering and functional decline, independently of whether or not they develop psychosis (1, 2);
2. The empirical evidence that the majority of subjects with a first-episode of psychosis were not previously intercepted in prodromal CHR-P stages through EIS (3, 4);
3. The lack of evidence on pharmacological and non-pharmacological interventions able to prevent the transition to psychosis from at-risk stages (5, 6).

On the background of these critiques and underlying a possible, unnecessary stigma related to the communication of a risk for psychosis, some authors proposed to abandon the current EIS model (7, 8). Alternatively, these authors support a shifting in the focus of intervention toward primary preventive interventions for the general population, aimed at reducing environmental risk factors (such as social marginality), early adverse experiences, and substance use. In our opinion, the unnecessary risk of stigmatization related to the communication of a risk for psychosis might be reconsidered; indeed, EIS usually intercept help-seeking young subjects that in the majority of cases persist in a state of psychological suffering and functional decline, in most cases requiring pharmacological interventions on top of psychosocial treatment, independently from the psychometric transition to psychosis (9).

## Clinical high-risk for psychosis as index of trans-prognostic severity

In this perspective, there is an increasing awareness that attenuated or intermittent psychotic symptoms in adolescence and youth represent a trans-diagnostic red flag of clinical severity, which might not be particularly specific with respect to the diagnostic outcome (e.g., affective vs. non-affective psychosis), but widely sensitive and reliable in terms of predicting the severity and persistence of clinical caseness (i.e., psychological suffering, functional decline, increased risk of biopsychosocial chronicity) and related need of care (10). Moreover, EIS can act as a structural bridge in the therapeutic management across the well-known “death valley” of continuity of care, i.e., the organizational hiatus between child- adolescent and adult mental health services. Such organizational compartmentalization intervenes precisely in that developmental phase between adolescence and young adulthood when the epidemiological risk for psychopathological manifestations peaks (11).

Therefore, to avoid the risk of prematurely “throwing the baby out with the bathwater” rather than abandoned, current EIS habits and structures could be revised and refreshed, along the general framework of transitional age/Youth Mental Health (YMH) (12). Furthermore, this is coherent with a more realistic, systemic, and clinically sensitive understanding of current evidence in the field. Indeed, the original mono-directional, homotypic view of the transition from CHR-P to psychosis (motivating the current EIS main focus on psychosis) gradually receded toward a more nuanced view of CHR-P as a pluripotential stage for multiple, heterotypic psychopathological outcomes, not limited to psychosis. Such broader, somehow more fluid view of multiple, non-deterministic trajectories plastically emerging from early, relatively unspecific and attenuated symptom networks in youth and, then, gradually evolving to more structured (and diagnostically characterized) symptom patterns in adulthood, is strongly aligned with real-world clinical practice.

## Empowering youth mental health: Phenomenological, developmental and transitional add-ons

Such a transformative, YMH-oriented view could be further reinforced by integrating phenomenological (13) and developmental perspectives (14), which would be beneficial both in terms of clinical depth (i.e., precision) and width (i.e., sensitivity). Indeed, the phenomenological perspective may counterbalance (or at least mitigate) the subtle risk of dimensional oversimplification, which is immanent to any overreliance on the trans-diagnostic status of certain symptom constructs. This is the case of the widespread, implicit assumption that psychotic subjective experience is a unitary condition, whose forms and contents are indistinguishable across trajectories headed toward schizophrenia, unipolar or bipolar depression, post-traumatic disorders, personality disorders, organic dementias, or substance abuse (15). A suitable clinical exploration of the experiential background from which psychotic symptoms emerge could be extremely informative: changes in the lived experience of time, space, self, and immediate immersion in the world, which inform psychotic phenomena in the schizophrenic spectrum are rather distal from (i.e., clinically difficult to confuse with) those characterizing vulnerability to mood disorders or dementia (16). Therefore, adding phenomenological depth could empower a YMH-oriented approach by enriching clinical formulation and further increasing precision (e.g., timely differential diagnosis and prognostic stratification). In parallel, overlaying a developmental perspective on transdiagnostic clinical stages might help reformulating and better understanding early biopsychosocial factors involved in the emergence of need of care and, thus, facilitate the accurate mapping of preclinical, developmental antecedents of broad psychopathological vulnerabilities, particularly in individuals at familial high risk (17).

A third aspect worth considering to accelerate the move toward YMH-oriented EIS is a deeper understanding of the “death valley” of continuity of care, which is due to the current, internationally widespread organizational hiatus between mental health services for children and adolescents and for adults (11). Transition psychiatry/mental health refers precisely to the multi-problematic issues related to the transfer from adolescent to adult care. Indeed, such historical division cuts across the youth age, when risk for mental disorders peaks (with obvious consequences in terms of undertreatment, discontinuity of care, and unmet needs), and this is perhaps the hardest obstacle against the implementation of 12-25 early intervention services. Indeed, this obstacle is not merely organizational but also cultural. Child and adolescent psychiatrists are used to dealing with plasticity and fluidity of childhood neurodevelopmental conditions that may follow heterotypic trajectories toward adolescence (e.g., from ADHD to mood disorders) as well as complex and multi-domain psychopathological presentation in adolescence, progressively taking a more precise shape in early adulthood. This is what has been increasingly reported in the current pandemic era (18), with adolescents presenting psychiatric emergencies characterized by constellations of mood, psychotic, dissociative and behavioral symptoms such as eating disorders, self-injuring, and social withdrawal. Alternately, adult psychiatrists are more often confronted with diagnostically clearer and stable conditions, which, once assessed, are generally canalized into treatment silos. Personality disorders are a glaring example of such substantial difference in the approach: they are indeed not easily ascertainable through the changeable behavioral patterns of adolescents, while they appear more clear-cut in adults.

## Conclusions

In sum, due to its conceptual history (i.e., a retrospective operationalization of prodromal symptoms of psychosis) the original CHR-P model was mainly viewed through homotypic lenses (i.e., from attenuated or intermittent to full-blown psychosis) which are generally more familiar to mental health services for adults (19). Almost three decades of CHR-P services implementation, however, clearly showed that homotypic trajectories from emerging to structured psychopathologies represent a substantial minority, whereas heterotypic trajectories are more frequent.

In conclusion, the implementation of YMH EIS may be an effective bridge between child-adolescent and adult mental health services constituting a catalyzing, transformative balance between them. Ideally, this would take the shape of a low-threshold and friendly, a(dia)gnostic entry point that progressively offers a gradient of more specific biopsychosocial interventions addressing emerging needs.

## Author contributions

MP: writing, original draft preparation, and conceptualization. AP and AR: conceptualization, writing, supervision, methodology, reviewing, and validation. All authors contributed to the article and approved the submitted version.

## Conflict of interest

The authors declare that the research was conducted in the absence of any commercial or financial relationships that could be construed as a potential conflict of interest.

## Publisher's note

All claims expressed in this article are solely those of the authors and do not necessarily represent those of their affiliated organizations, or those of the publisher, the editors and the reviewers. Any product that may be evaluated in this article, or claim that may be made by its manufacturer, is not guaranteed or endorsed by the publisher.
